# Gray matter covariations and core symptoms of autism: the EU-AIMS Longitudinal European Autism Project

**DOI:** 10.1186/s13229-020-00389-4

**Published:** 2020-10-30

**Authors:** Ting Mei, Alberto Llera, Dorothea L. Floris, Natalie J. Forde, Julian Tillmann, Sarah Durston, Carolin Moessnang, Tobias Banaschewski, Rosemary J. Holt, Simon Baron-Cohen, Annika Rausch, Eva Loth, Flavio Dell’Acqua, Tony Charman, Declan G. M. Murphy, Christine Ecker, Christian F. Beckmann, Jan K. Buitelaar, the EU-AIMS LEAP group

**Affiliations:** 1 Department of Cognitive Neuroscience, Donders Institute for Brain, Cognition and Behaviour, Radboud University Nijmegen Medical Centre, Nijmegen, The Netherlands; 2 Karakter Child and Adolescent Psychiatry University Centre, Nijmegen, The Netherlands; 3 Department of Psychology, Institute of Psychiatry, Psychology and Neuroscience, King’s College London, London, UK; 4 Department of Psychiatry, Brain Center Rudolf Magnus, University Medical Center Utrecht, Utrecht, The Netherlands; 5 Department of Psychiatry and Psychotherapy, Central Institute of Mental Health, Medical Faculty Mannheim, University of Heidelberg, Mannheim, Germany; 6 Department of Child and Adolescent Psychiatry, Central Institute of Mental Health, Medical Faculty Mannheim, University of Heidelberg, Mannheim, Germany; 7 Autism Research Centre, Department of Psychiatry, University of Cambridge, Cambridge, UK; 8 Department of Forensic and Neurodevelopmental Sciences, Institute of Psychiatry, Psychology and Neuroscience, King’s College London, London, UK; 9 Department of Child and Adolescent Psychiatry, University Hospital, Goethe University, Frankfurt am Main, Germany; 10 Centre for Functional MRI of the Brain, University of Oxford, Oxford, UK

**Keywords:** Autism, Magnetic resonance imaging, Voxel-based morphometry, Independent component analysis, Canonical correlation analysis

## Abstract

**Background:**

Voxel-based morphometry (VBM) studies in autism spectrum disorder (autism) have yielded diverging results. This might partly be attributed to structural alterations being associating with the combined influence of several regions rather than with a single region. Further, these structural covariation differences may relate to continuous measures of autism rather than with categorical case–control contrasts. The current study aimed to identify structural covariation alterations in autism, and assessed canonical correlations between brain covariation patterns and core autism symptoms.

**Methods:**

We studied 347 individuals with autism and 252 typically developing individuals, aged between 6 and 30 years, who have been deeply phenotyped in the Longitudinal European Autism Project. All participants’ VBM maps were decomposed into spatially independent components using independent component analysis. A generalized linear model (GLM) was used to examine case–control differences. Next, canonical correlation analysis (CCA) was performed to separately explore the integrated effects between all the brain sources of gray matter variation and two sets of core autism symptoms.

**Results:**

GLM analyses showed significant case–control differences for two independent components. The first component was primarily associated with decreased density of bilateral insula, inferior frontal gyrus, orbitofrontal cortex, and increased density of caudate nucleus in the autism group relative to typically developing individuals. The second component was related to decreased densities of the bilateral amygdala, hippocampus, and parahippocampal gyrus in the autism group relative to typically developing individuals. The CCA results showed significant correlations between components that involved variation of thalamus, putamen, precentral gyrus, frontal, parietal, and occipital lobes, and the cerebellum, and repetitive, rigid and stereotyped behaviors and abnormal sensory behaviors in autism individuals.

**Limitations:**

Only 55.9% of the participants with autism had complete questionnaire data on continuous parent-reported symptom measures.

**Conclusions:**

Covaried areas associated with autism diagnosis and/or symptoms are scattered across the whole brain and include the limbic system, basal ganglia, thalamus, cerebellum, precentral gyrus, and parts of the frontal, parietal, and occipital lobes. Some of these areas potentially subserve social-communicative behavior, whereas others may underpin sensory processing and integration, and motor behavior.

## Background

Autism spectrum disorder (henceforth autism) is an early onset neurodevelopmental condition characterized by core deficits in social interaction and communication, along with restrictive interests and behavior, and sensory abnormalities [1]. Magnetic resonance imaging (MRI) studies have increased our understanding of the neuroanatomical underpinnings of autism and show that autism is associated, at the group level, with brain structural changes [2]. However, many results are not robust across different studies. For example, two studies using the same large-scale open access Autism Brain Imaging Data Exchange (ABIDE) dataset [3] came to different conclusions with regard to the volume of the pallidum [4, 5]. Also, across whole brain approaches investigating cortical (i.e., cortical thickness and surface area) and subcortical (i.e., volume) features have been inconsistent; two large-scale pooled estimate analytical studies observed diverging results of cortical changes in autism [6, 7]. Similarly, autism studies quantifying voxel-wise gray matter (GM) density also found divergent results of GM differences between autism diagnosed and control individuals; for instance, meta-analyses reported diverse changes of GM morphometry in autistic individuals on average, reporting either increased or decreased density of right inferior temporal gyrus in autism [8, 9]. Even when taking age into account, studies still observed different structural brain alterations in children and adolescents with autism [10, 11].

A commonality to all these studies is their reliance on mass-univariate statistics. This approach identifies alterations in isolated regions or voxels but ignores possible relationships between them. The brain is a complex system of interconnected networks, and research into the neural basis of autism has moved away from focusing on local abnormalities into conceptualizing autism as a disorder of alterations in structural and functional brain connectivity [12]. This implies that structural brain alterations in autism likely reflect the combined influence of several regions and are not confined to one specific region [13, 14]. The present paper aims to advance prior work on brain structural neural correlates of autism in two ways. First, we aim to move away from the standard univariate approach and incorporate an alternative that adheres more closely to the hypothesis of autism as a disconnection syndrome [13], thus providing greater sensitivity for between-group effects. For this purpose, we identify inter-regional sources of structural covariation using independent component analysis (ICA) [15], a data-driven unsupervised approach that allows the identification of interconnected brain regions across the whole brain. It has previously been applied successfully to identify covariance of brain morphometry in patients with psychiatric disorders [16–18]. Second, we move beyond the categorical autism case–control comparison towards exploring associations between brain structure and symptom dimensions or profiles of autism. Although former studies have used univariate approaches to explore the relationship between brain substrates and clinical phenotypes [6, 19], such associations are potentially the consequence of integrated effects across multiple symptoms dimensions and brain regions, rather than simple associations between a specific brain region and a specific symptom dimension. To study such multidimensional associations multivariate methods are effective [20, 21] and here we achieve this integration using Canonical Correlation Analysis (CCA) [22].

In summary, we investigate alterations in GM morphometric covariations in a deeply phenotyped large European autism case–control sample [23, 24] that allows us to improve our understanding of correlated structural brain alterations in autism. Subsequently, we focus on the covariation between the identified structural features and symptom behavior profiles among individuals with autism.

## Methods

### Participants

The participants were selected from the first wave of the European Autism Interventions—A Multicentre Study for Developing New Medications (EU-AIMS) Longitudinal European Autism Project (LEAP) dataset, which is a large multicenter study that aims to identify and validate biomarkers for autism [24]. In total, six centers are involved: Institute of Psychiatry, Psychology and Neuroscience, King’s College London, United Kingdom; Autism Research Centre, University of Cambridge, United Kingdom; Radboud University Medical Centre, Nijmegen, the Netherlands; University Medical Centre Utrecht, the Netherlands; Central Institute of Mental Health, Mannheim, Germany; and University Campus Bio-Medico, Rome, Italy. Each participant underwent clinical, cognitive, and MRI assessment. Autism diagnoses were confirmed by clinicians according to the Diagnostic and Statistical Manual-IV (DSM-IV), International Statistical Classification of Diseases and Related Health Problems 10th Revision (ICD-10), or DSM-5. The study was approved by local ethical committees in each participating center, and written informed consent was provided by all participants and/or their legal guardians (for those < 18 years old). For further details on experimental design and clinical assessments, see [23, 24].

In the present study, we selected participants with available structural MRI data. All images were inspected visually and participants were excluded in cases of brain injury or structural abnormalities (e.g., enlarged ventricles or cysts), excessive head motion, or preprocessing failure (*n* = 29). We excluded the participants from the Rome site due to low sample size (*n* = 1). We also excluded participants without full-scale intelligence quotient (FSIQ) data in the further statistical analyses (*n* = 5). This resulted in a sample of 599 participants from 5 sites, including 347 individuals with autism and 252 typically developing (TD) controls. Demographic and clinical information is shown in Table 1.

**Table 1:** Participant characteristics

Demographic	Autism, *n* = 347		TD, *n* = 252		*t*/χ^2^	*p* value
	Mean	SD	Mean	SD		
Age, years^a^	16.79	5.56	16.92	5.71	− 0.270	0.788
FSIQ^a,b^	99.40	18.94	104.88	18.26	− 3.549	*p* < 0.001
FSIQ ≥ 75	104.29	14.95	109.02	13.07	− 3.883	*p* < 0.001
FSIQ < 75	66.61	5.44	63.69	9.20	1.399	0.172

	*n*	%	*n*	%		
Sex, male/female^c^	253/94	72.9/27.1	163/89	64.7/35.3	4.658	0.031
ADHD rating scale^d^, with/without ADHD	139/160	46.5/53.5	21/180	10.4/89.6	71.750	*p* < 0.001

Symptom profiles	Mean	SD	Mean	SD		
ADI^e^						
Social interaction	16.80	6.66				
Communication	13.50	5.62				
RRB	4.32	2.67				
ADOS^f^						
Social affect	6.04	2.59				
RRB	4.73	2.78				
SRS T-score^g^	70.59	12.06	47.24	8.79		
RBS^h^	15.76	13.42	2.20	8.28		
SSP^i^	138.62	27.28	175.97	16.18		

TD, typically developing; SD, standard deviation; FSIQ, full-scale intelligence quotient; ADHD, Attention Deficit Hyperactivity Disorder; ADI, Autism Diagnostic Interview-Revised; RRB, restricted, repetitive behaviors; ADOS, Autism Diagnostic Observational Schedule 2; SRS, Social Responsiveness Scale 2nd Edition; RBS, Repetitive Behavior Scale-Revised; SSP, Short Sensory Profile

^a^Statistical differences were assessed by two-sample *t* test

^b^In Schedule A, B, and C (FSIQ ≥ 75), there are 302 participants with autism and 229 participants with TD. Schedule D (FSIQ < 75) comprised 45 participants with autism and 23 TD individuals

^c^Sex difference was examined by the chi-square test

^d^ADHD rating scores were available for 500 participants, including 299 individuals with autism and 201 TD individuals

^e^ADI scores were available for 332 participants

^f^ADOS scores were available for 339 participants. We considered calibrated severity scores

^g^Parent report SRS scores were available for 284 participants with autism and 135 TD individuals

^h^RBS scores were available for 277 participants with autism and 133 TD individuals

^i^SSP scores were available for 201 participants with autism and 115 TD individuals

^g,h,i^In all questionnaires, the scores of the autism group only were used in our study, and they are all parent-rated

### Clinical measures

We used the Autism Diagnostic Interview-Revised (ADI) [25] and the Autism Diagnostic Observational Schedule 2 (ADOS) [26] to quantify past (ever and previous 4-to-5 years) and current autism symptoms of the social interaction, communication, and restricted repetitive behaviors (RRB) domains. We used T-scores (age- and sex-adjusted) of the Social Responsiveness Scale 2nd Edition (SRS) [27] in the autism group to assess severity of autistic traits/symptoms and the Repetitive Behavior Scale-Revised (RBS) [28] to measure repetitive and rigid behaviors associated with autism. Moreover, sensory processing abnormalities of autism were assessed with the Short Sensory Profile (SSP) [29]. To examine associations between clinical features in autism and brain measures, we created two sets of clinical measures: (1) the subscale scores of ADI-R and ADOS, both instruments were rated by qualified examiners, and (2) the total scores of SRS, RBS, and SSP, we included parent-rated reports only and limited the analyses to within the autism group. Further, concerning the potential effect of comorbidity with Attention Deficit Hyperactivity Disorder (ADHD), we included comorbidity with ADHD as an additional covariate in analyses. ADHD symptoms were assessed with the ADHD DSM-5 rating scale that includes symptom scales of inattention and hyperactivity/impulsivity symptoms. The ADHD DSM-5 rating scale was based on either parent-report or self-report scores; self-report scores were only used when parent-reports were unavailable. The categorical output of the ADHD rating scale was used in this study. The summary for each of these clinical measures can be found in Table 1.

### MRI data acquisition

All participants were scanned on 3 T MRI scanners (University of Cambridge: Siemens Verio; King’s College London: GE Medical Systems Discovery MR 750; Mannheim University: Siemens TimTrio; Radboud University: Siemens Skyra; Rome University: GE Medical Systems Sigma HDxTt; Utrecht University: Philips Medical Systems Achieva/Ingenia CX). High-resolution structural T1-weighted images were acquired with full head coverage, at 1.2 mm thickness with 1.1 × 1.1 mm in-plane resolution. For all other scanning parameters, please see Additional file 1: Subsection 1.

### GM density estimation

Voxel-based morphometry (VBM) is a spatially unbiased whole-brain approach that extracts voxel-wise GM density (the amount of GM at a voxel) estimations. We performed VBM analyses using the CAT12 toolbox [30] in SPM12 (Wellcome Department of Imaging Neuroscience, London, UK). T1-weighted images were automatically segmented into GM, white matter, and cerebrospinal fluid and affine registered to the MNI template to improve segmentation. All resulting segmented GM maps were then used to generate a study-specific template and registered to MNI space via a high-dimensional, nonlinear diffeomorphic registration algorithm (DARTEL) [31]. A Jacobian modulation step was included using the flow fields to preserve voxel-wise information on local tissue volume. Images were smoothed with a 4 mm full-width half-max (FWHM) isotropic Gaussian kernel.

A full quality control report was generated by the CAT SPM pipeline for each participant that included visualizations of the segmentation, which were evaluated visually, and quantitative quality measures including mean correlation from sample homogeneity module and weighted overall image quality ratings that were additionally used to detect and exclude images of insufficient quality for inclusion in analysis. We visually checked the images with the mean correlation smaller than three standard deviations from the sample mean. Accordingly, 5 participants required visual inspection after preprocessing, whereas none of them were observed obvious artifacts, and they were consequently included in the final analyses.

### Structural ICA decomposition

All participants’ VBM data were simultaneously decomposed into 100 spatially independent sources of spatial variation using MELODIC-ICA [15]. Such ICA decomposition provides, at each independent component, a brain map reflecting a pattern of GM density covariation across participants, and a participant’s loading vector reflecting the contribution of each participant to each component. Higher model orders (i.e., number of components) tend to extract spatially sparser maps, and hence participant loading vectors variance would be explained by smaller subsets of participants. Therefore, 100 components were chosen to capture as much variation as possible while remaining statistically powered. However, the choice of model order is heuristic, we thereby examined the dependence on model order using different dimensionalities; more precisely, in addition to the 100 dimensional factorization, we also considered an automatic dimension estimation approach as implemented in MELODIC-ICA and a 50 dimensional independent component factorization.

### Statistical approach

For completeness we first performed a standard mass-univariate statistical analyses directly on the GM densities. To that end we used a generalized linear model (GLM) to detect group differences (autism vs. TD) on GM densities using the FMRIB Software Library v6.0 (FSL) [33]. Participants’ VBM maps were considered as the dependent variable and diagnostic group as the independent factor, with age, sex, FSIQ, and scan site as covariates. Significance was assessed using permutation testing (5000 permutations) and correction for multiple comparisons was achieved using Threshold-Free Cluster Enhancement (TFCE, two-tailed, threshold at *p* < 0.05) [34, 35].

Next, we considered the results of the ICA factorization of the VBM data. A GLM was used to examine differences between autistic and TD individuals, by using each participant's loading to each component as a dependent variable, diagnostic group as independent variable, and age, sex, FSIQ, and scan site as regressors. To avoid the results of case–control differences being biased by IQ, we repeated the same procedure by excluding participants in Schedule D (FSIQ < 75, more details see Additional file 1: Subsection 2). Considering that autism is highly comorbid with ADHD [36], we also controlled for comorbidity with ADHD by adding a dummy-coded variable (with/without ADHD) to the original GLM analyses (autism vs. TD) in the case–control ICA analysis. Additionally, we also conducted analyses that included a dimensional score (instead of categorical code) of the two ADHD subscales as an additional covariate in the group effect analyses (see Additional file 1: Subsection 3). Furthermore, we separately checked the effects of age squared, age-by-group, age squared-by-group, sex-by-group interactions, anxiety, depression comorbidity, medication use and image quality on case–control differences of structural covariance. The results can be found in Additional file 1: Subsection 3. Multiple comparison correction was implemented using false discovery rate (FDR) (*p* < 0.05) [37].

We also explored independently the relationships between each estimated brain component and subscales of ADI and ADOS, SRS, RBS, and SSP in the autism group using GLM analyses and again correction for multiple comparisons was implemented with the FDR method (*p* < 0.05). Then, to simultaneously explore the relationship between all the brain structural phenotypes estimated through ICA and all symptom phenotypes in the autism group, we used CCA. In the considered scenario, CCA is able simultaneously to learn linear projections (via canonical coefficients) of the brain structural sources and the behavioral measures that maximize the correlation between them at the participant level. Canonical variates are generated independently for the brain and the behavioral datasets according to the product of the canonical coefficients (learned through CCA) and the original datasets. We referred to each pair of canonical variates as CCA mode [38]. The canonical coefficients separately represent weights for each brain variable and each symptom measure. For interpretation of the contribution of each independent source and each clinical measure to the CCA mode, their canonical coefficients were corrected as noted in [39]. In this work we focused on the first canonical mode since it provides the strongest associations and we denoted it as the *main CCA mode*. Furthermore, for each CCA analysis the statistical significance of the main CCA mode was determined by permutation testing (10,000 permutations, Bonferroni corrected *p* < 0.05/number of CCA modes). Here, we performed two separate CCA analyses to link the independent components participants’ contributions to subsets of behavioral measures; in the first CCA analyses (CCA_1_) we included the subscales of ADI and ADOS as clinical measures and in the second (CCA_2_) we used total scores of SRS, RBS, and SSP. The reliability of the CCA results presented as well as its dependence on the number of participants were tested using a leave-one-out cross-validation approach (Additional file 1: Subsection 4).

## Results

### Mass-univariate statistics

The standard mass-univariate GLM analysis of the VBM data comparing cases and controls did not show significant group differences for voxel-wise GM volumes after multiple comparison correction. We provide the uncorrected statistical results (*p* < 0.05) in Additional file 1: Subsection 5.

### Group effect on ICA decomposition

The structural data ICA decomposition provided a set of 100 independent spatial sources, each of which is connected to a vector that depicts the degree of each participant’s contribution to the corresponding components. For clarity, we further refer to these vectors as the participant loadings. Post-hoc GLM analyses of these participant loadings showed case–control differences at nine independent components (ICs) (*p* < 0.05, i.e., IC10, IC13, IC14, IC15, IC23, IC28, IC31, IC48, and IC 99, see Additional file 1: Subsection 6). Of these, two components, IC10 ($\beta = - 0.147$, *p* = 8.850 × 10^–5^, effect size [Cohen’s d] *d* = − 0.358) and IC14 ($\beta = - 0.132$, *p* = 5.450 × 10^–4^, *d* = − 0.321), survived multiple comparison correction (FDR corrected, *p* < 8.072 × 10^–4^). These results were not driven by age, sex, or scan site.

**Fig. 1:**
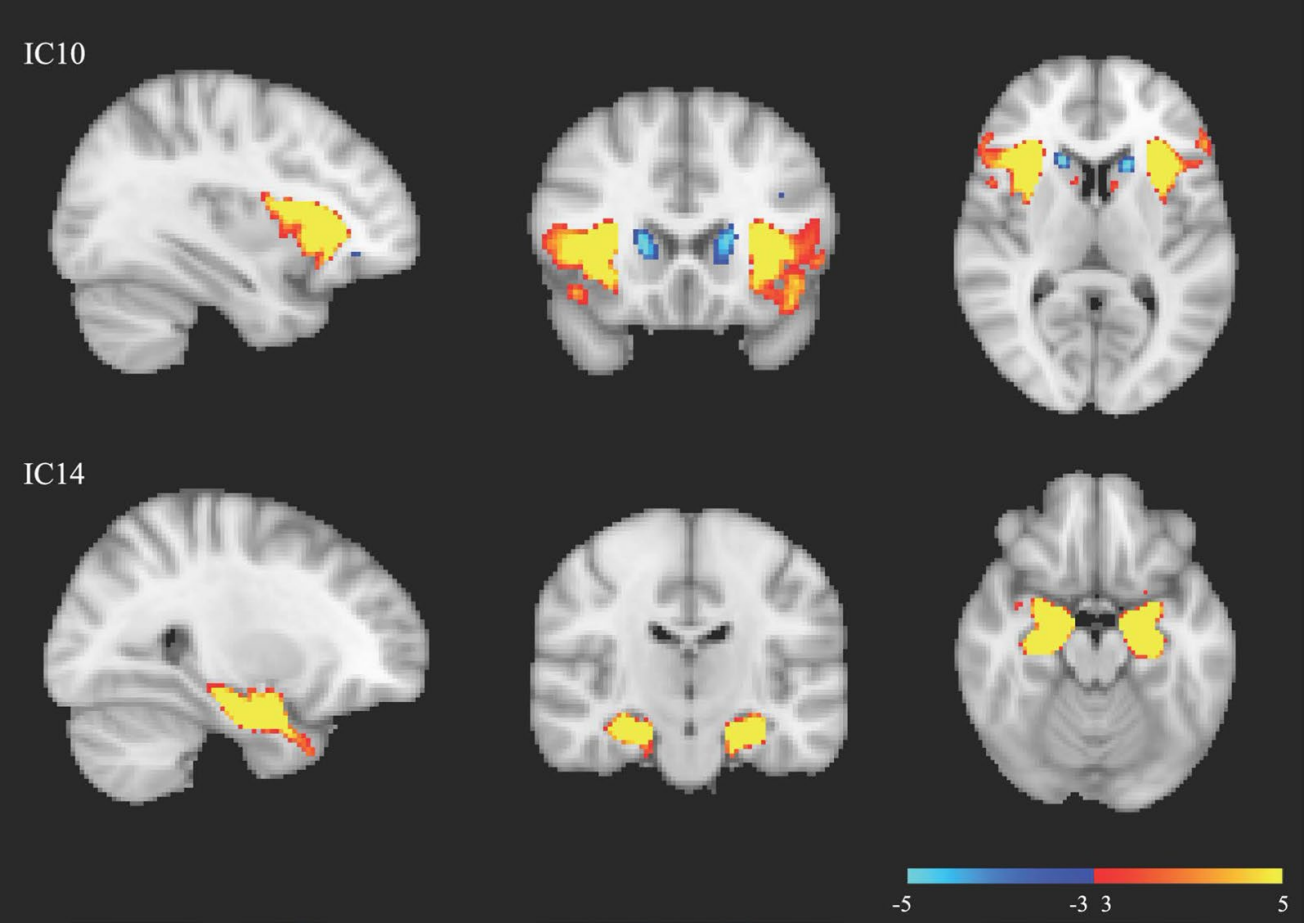
The components showed significant case–control differences. The component maps were thresholded at $3 < |Z| < 5$. IC10, component number 10; IC14, component number 14

In Fig. 1, we present summary images reflecting the brain areas involved in the structural variances occurring at these two components. The top row of Fig. 1 shows that IC10 primarily relates to structural variation in the bilateral insula, inferior frontal gyrus (IFG), orbitofrontal cortex (OFC), and caudate nuclei. Among these brain regions, the bilateral caudate exhibits alterations in the opposite direction to the others. Given the negative beta coefficient obtained from the GLM analysis between participant loadings at IC10 and the diagnosis group labels, individuals with autism demonstrate increased GM densities in the bilateral caudate and decreased densities in the bilateral insula, IFG, and OFC. The bottom row of Fig. 1 shows that IC14 mainly involves variations in the bilateral amygdala, hippocampus, and parahippocampal gyrus (PHG). Similarly, according to the sign of the beta values obtained through the GLM, the autism group shows decreased densities in the areas involved in IC14.

The robustness of the ICA results to the model order choice was evaluated by considering, in addition to the original 100-dimensional factorization, an automatic dimensionality estimation procedure resulting in a 91-dimensional factorization, and a 50-dimensional factorization. We observed that the main components reported (IC10 and IC14) are highly reproducible independent of the model order choice. For details, see Additional file 1: Subsection 7.

To validate the ICA results not being biased by low IQ participants, an additional validation was performed by taking FSIQ into account to exclude the participants in Schedule D from the ICA factorization. This showed that a unique IC, corresponding to the original IC10, survived FDR correction (see Additional file 1: Subsection 3). Further, the effect of comorbidity with ADHD on brain structural variations was determined using data from 500 participants (for detailed demographic information, see Additional file 1: Subsection 3). This analysis showed that IC14 remained significantly associated with the autism group (FDR corrected, *p* = 9.669 × 10^–4^). However, IC10 was no longer associated with autism (*p* = 0.004).

Further, post-hoc GLM analyses of the relationships between brain ICs and symptom ratings did not provide any significant associations (Additional file 1: Subsection 8).

### Relating gray matter spatial variation patterns to symptoms profiles

**Fig. 2:**
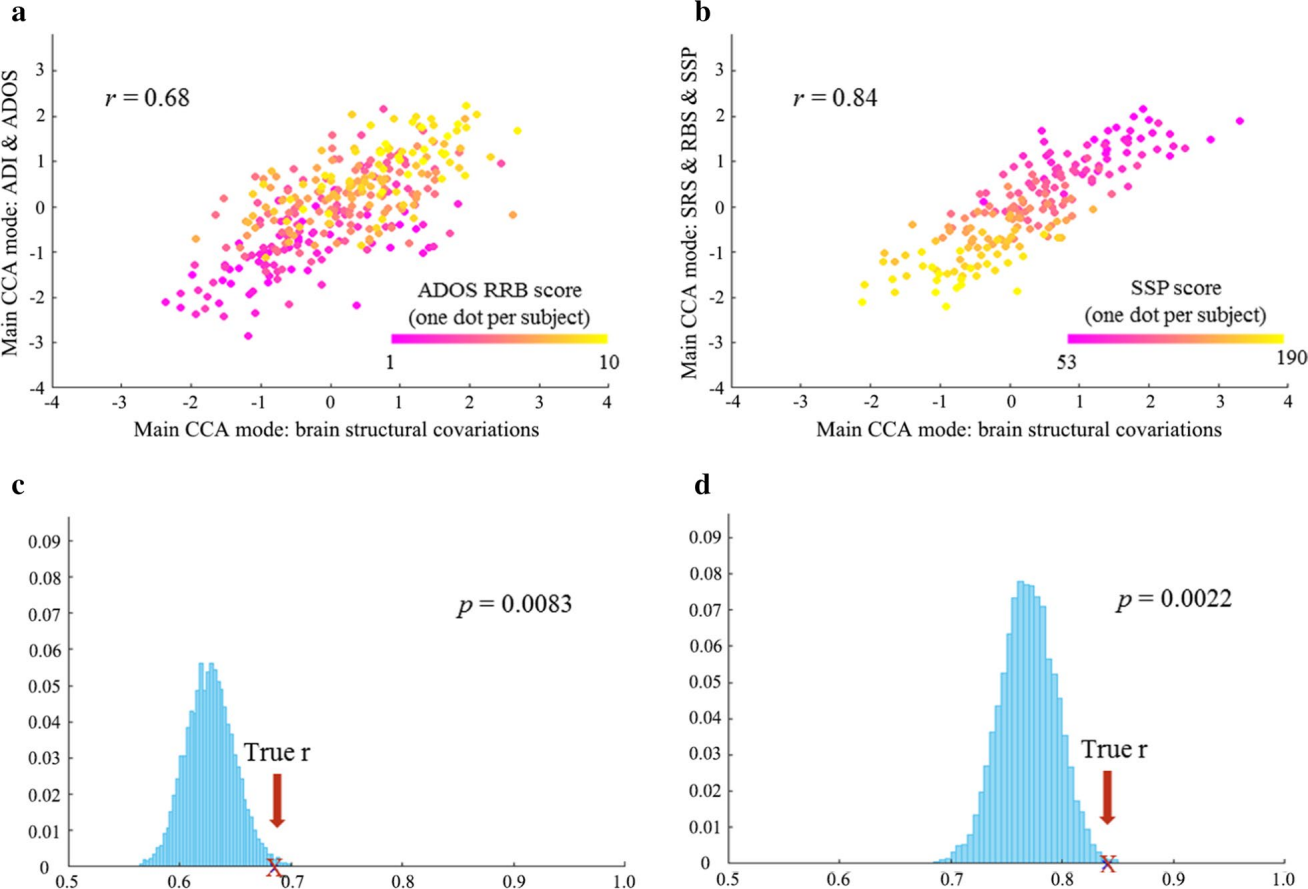
The first row shows the scatterplot of the main CCA mode of the brain structural covariations versus the symptom profiles for CCA_1_ and CCA_2_ respectively. One dot per participant in each graph is coded with gradient colors according to the scores of ADOS RRB (**a**) and SSP (**b**), respectively. The second row shows the histograms of the null distribution of correlation values obtained from the main CCA mode at 10,000 random participants’ permutations in the autism sample with ADI and ADOS scores (**c**), and with SRS, RBS, and SSP scores (**d**). The true *r*-value is marked by a red cross. ADI, Autism Diagnostic Interview-Revised; ADOS, Autism Diagnostic Observational Schedule 2; SRS, Social Responsiveness Scale 2nd Edition; RBS, Repetitive Behavior Scale-Revised; SSP, Short Sensory Profile; RRB, restricted and repetitive behaviors

**Fig. 3:**
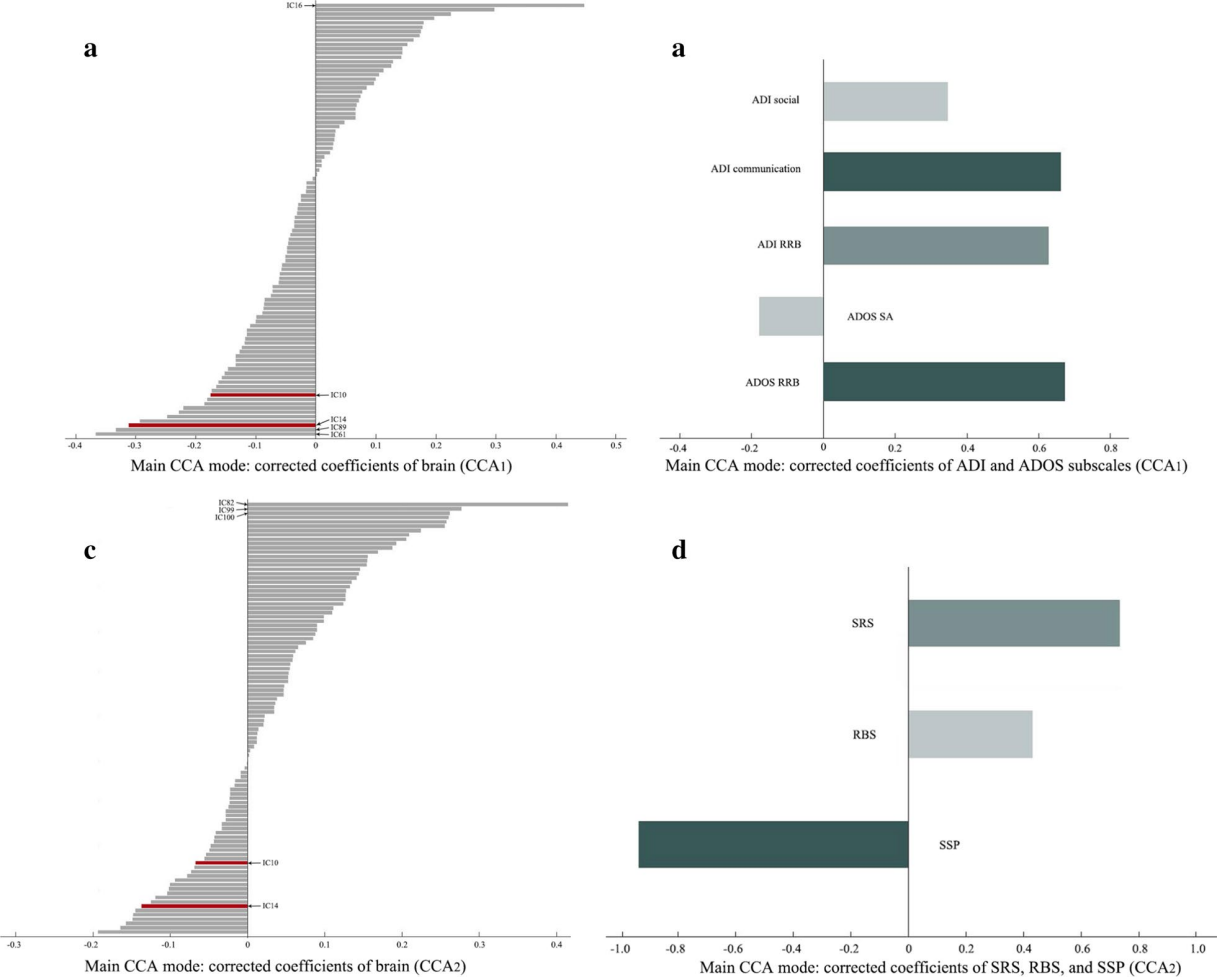
The top row shows the corrected canonical coefficients (weights) of the main CCA mode for the CCA_1_ analyses (ADI&ADOS), and the bottom row for the CCA_2_ analyses (SRS&RBS&SSP). **a**, **c** display the degree that each brain component contributed to the main CCA mode in each analysis. The two components with significant group effects are displayed in red. **b**, **d** display the degree that each symptom profile contributes to each analysis. CCA, canonical correlation analysis; ADI, Autism Diagnostic Interview-Revised; ADOS, Autism Diagnostic Observational Schedule 2; SRS, Social Responsiveness Scale 2nd Edition; RBS, Repetitive Behavior Scale-Revised; SSP, Short Sensory Profile

As a final step, we applied CCA to examine the associations between the 100 components and the two sets of clinical measures among the autism cases only. The CCA_1_ (linking ADI and ADOS subscale scores to brain measures), involved 325 autism participants and showed a Bonferroni corrected (*p* = 0.05/5 = 0.010) significant relationship (Fig. 2a,c, *r* = 0.701, permutation *p* = 0.008). In this main CCA mode, IC16, IC61, IC89, and IC14 were the highest contributors to the correlation with autism symptoms, and the ADOS RRB subscale loaded most on the association with the brain measures (Fig. 3a, b). Among the four components, IC16 mainly involved density variations in bilateral thalamus and putamen (canonical weight: 0.447), IC61 in right lateral occipital and left superior parietal lobe (canonical weight: − 0.366), and IC89 in the right precentral gyrus (canonical weight: − 0.333). For details, see Additional file 1: Subsection 9. Note that IC14 is among the components previously reported showing linear significant case–control group effects. The regions involved in IC14 were mentioned above (canonical weight: − 0.312). Since higher scores of the ADI and ADOS reflect more severe autism symptoms, positive values of IC16 suggest that higher loading on this component is related to more severe symptoms in autism, and negative values of IC61, IC89, and IC14 meant that lower loadings on these three ICs are associated with more severe symptoms. In Fig. 2a, participants were color coded according to their ADOS-RRB scores to illustrate how the ADOS-RRB score drives the canonical correlation.

In CCA_2_ we linked SRS, RBS, and SSP scores to the brain measures of 194 individuals with autism, which is 55.9% of all participants with autism (lower number due to missing questionnaire data). We found a Bonferroni corrected (*p* = 0.05/3 = 0.017) significant relationship (Fig. 2b, *r* = 0.840, permutation *p* = 0.002, Fig. 2d). In this main CCA mode, IC82, IC99, and IC100 were the highest contributors to the correlation with behavior profiles, and SSP score loaded most on the association with the brain measures in the autism group (Fig. 3c, d). IC82 mainly involved variations in the bilateral cerebellum (canonical weight: 0.414), IC99 in the left lateral occipital and parietal lobe, and bilateral precentral gyrus (canonical weight: 0.277), and IC100 in the left inferior frontal gyrus and right middle frontal lobe (canonical weight: 0.262). For details, see Additional file 1: Subsection 9. Similarly, lower loadings on these three ICs were related to more severe symptoms. In Fig. 2b, each participant was color coded according to their SSP score, and it shows how SSP score drives the correlation. In this case, both IC10 and IC14 ranked outside the top 20 of the 100 components, suggesting that these two components with significant case–control difference have no strong contribution to the CCA_2_ correlation. However, for completeness, direct interpretation (referring to uncorrected coefficients) of the CCA_2_ weights ranks IC14 as the third strongest contributor to this canonical correlation (Additional file 1: Subsection 10).

The CCA robustness analyses indicated that the main CCA modes of both CCA analyses were reliably estimated in a leave-one-subject out setting (Additional file 1: Subsection 4). In CCA_1_, the weights of the main CCA mode of each leave-one-out analysis correlated on average above 0.94 with the weights of original main CCA mode in brain loadings and above 0.95 in behavior phenotypes when the sample was bigger than 122 participants. In CCA_2_, the weights of the main CCA mode related on average above 0.92 in brain loadings and above 0.96 in behavior profiles when the sample was bigger than 111 participants. Both CCA analyses are no reproducible for sample sizes smaller than (approximately) 100 participants.

## Discussion

The present study used a reliable approach to quantify inter-individual differences in GM morphometry covariations in a deeply phenotyped large sample of individuals with and without autism. The standard, univariate VBM analysis did not show significant case–control differences. We then utilized an ICA decomposition of all participants GM density images, and similarly performed a case–control post-hoc statistical analyses. This analysis showed that autism was significantly associated with alterations in two independent sources of GM density covariations. These findings corroborated our hypothesis that alterations in brain morphometry in autism are associated with the combined influence of several regions rather than with a single region. In a following step, we applied CCA to explore multivariate associations between sets of continuous measures of core symptoms and sets of ICA-derived morphometry measures within the autism group, and were able to identify significant relationships between brain components and symptom profiles. Notably, one of the components which showed significant case–control differences was also among the highest loading components in the CCA.

We found nonsignificant case–control difference in univariate analysis in current study, which is inconsistent with previous studies (e.g., [8, 9]). In light of the substantial biological heterogeneity among autistic individuals, it is expected that findings differ across datasets, especially those of smaller size. Specifically, the current study included the individuals with a relative wide age and IQ range, and did not exclude co-occurring psychiatric symptoms. Additionally, integrating the findings from multivariate analyses in our study, the absence of diagnostic differences at single region/voxel underscores the detection sensitivity of group effect of a multivariate approach which evidently verified our hypothesis.

Our findings showed two covarying sets of brain areas that structurally differed between cases and controls. While one source of GM density covariation, IC10, mainly related to the bilateral insula, IFG, OFG, and caudate, another source, IC14, primarily involved the bilateral amygdala, hippocampus, and PHG. The brain regions within each component are anatomically clustered and symmetrical, which indicates that the independent structural covariation alteration in the GM of individuals with autism is concentrated in nearby brain areas. This is in line with a previous study that used a similar approach [40]. It is further in line with organizing principles of the brain that regions tend to be more interconnected when they are located close to each other [41, 42]. However, when we compared the regions loading on the two components, the covarying regions of each component distribute in different brain locations. This suggests that neuroanatomic alterations underlying autism are more widely distributed at the whole brain level. It is of note that, when accounting for ADHD comorbidity, IC14 remained significant but IC10 did not. This suggests that IC14 is more specifically related to autism associated structural variations, even after linearly accounting for ADHD effects, while IC10 might reflect variations associated with both autism and ADHD.

The brain regions with high loadings on either of these two components, i.e., insula, amygdala, hippocampus and PHG have lower densities in autism and have earlier been associated with autism [9, 43]. The opposite direction of the alteration of the caudate nucleus in autism has also previously been found [44]. This is not the case for the IFG and OFG, which showed lower densities in autism in our study, where prior studies found mixed results [45, 46]. Importantly, the brain regions identified by our analyses have earlier been implicated in the neurobiology and/or neurocognition of autism. In IC10, structural and/or functional alterations of the insula, IFG, and OFC have been associated with social and non-social cognitive impairments in autism [46–49]. A meta-analysis reported abnormal functional activations of the insula, IFG, and OFG during social cognition tasks in autism [50]. Additionally, variance of the caudate nucleus volume was found to correlate with the severity of RRB symptoms in autism [44]. Together with deviant structural and functional connectivity between frontal cortical areas and striatum in autism [47, 51, 52], structural covariation in striatum and frontal areas may underlie atypical functional fronto-striatal connectivity, and this has been associated with repetitive behavior and executive functioning impairments in autism [3, 45]. In the present study, the density of caudate nuclei increase simultaneously with densities decreasing on other areas in autism, which fits with the results of a few functional studies that indicate inverse functional changes of these areas [53]. Particularly, the special pattern of GM densities changes in frontal and striatal areas might serve an important role in autism-related symptoms.

In IC14, we found decreased densities of amygdala and hippocampus, where the structural alterations have previously been related to social deficits in autism [6, 54]. The amygdala, hippocampus, and PHG subserve cognitive and emotional functions that were found abnormal in individuals with autism [50, 55, 56]. In addition to being involved in emotion and face processing, the three areas have been proposed as structures critical for working memory in autism [57]. Furthermore, these cognitive domains exert bidirectional effects on each other, with atypical social-emotional processing influencing memory performance in individuals with autism, and memory being involved in complex information processing and executive functioning, which in turn affects social cognition [57, 58]. In sum, given the potential functional interactions between these three brain areas, the structural covariance alterations of the amygdala, hippocampus, and PHG found in our study, may underlie or contribute to abnormal functional connections of these areas, and thus underlie poor performance on social cognition and memory tasks in individuals with autism.

Our multivariate correlation analyses moved from the case–control comparison to the use of continuous symptoms among individuals with autism and identified two prominent relationships between all structural brain covariances and symptoms in autism. Three of the four brain components that ranked top in this analysis did not show case–control differences, while there was one component (IC14) that differed between cases and controls and also significantly correlated with the severity of autism symptoms assessed by ADI and ADOS. The brain areas loading high on the brain components identified in the CCA are somewhat different from those implicated in the case–control analyses. These former brain areas are the thalamus, putamen, precentral gyrus, and parietal and occipital lobes in CCA_1_, and the cerebellum, frontal lobe, and again precentral gyrus, and parietal and occipital lobes in CCA_2_. These are foremost areas of the brain implicated in the processing and higher order integration of sensory information and motor functions. This makes sense since repetitive, rigid and stereotyped behaviors and abnormal sensory behaviors seem to drive the brain-behavior associations much more than the measures on social-communication symptoms. Note that variance within the different autism symptom domains (social-communication, repetitive behaviors and sensory abnormalities) was similar and cannot explain the differential symptom-brain associations.

Overall, the results of our multivariate analyses on case–control differences and on continuous measures of symptom severity among those with autism demonstrate the complexity of the brain morphometry correlates of autism. Brain areas involved are scattered across the whole brain and include the limbic system, basal ganglia, thalamus, cerebellum, precentral (motor) gyrus, and parts of the frontal, parietal, and occipital lobes.

## Strengths and limitations

The strengths of our study are the use of a large deeply phenotyped sample, bottom-up data-driven analyses, a multivariate approach for examining brain-symptom associations, and a large set of continuous measures of core autism symptoms. There are several limitations in our study. First, only 55.9% of the autism group had complete questionnaire data on continuous parent-reported symptom measures, which may have lowered statistical power for this analysis. Second, due to the clinical characteristic of autism, the participants showed significant differences in the proportion of sex distribution. Despite this we regressed out the sex effect in the group difference analyses, future studies may consider balancing the sex distributions in case and control groups; however this may be difficult to achieve. Third, albeit we ran a series of sensitivity and control analyses, the effect of other potential sources of variance, such as the complex effects of medication use that may influence our findings.

## Conclusions

We demonstrate brain morphometry differences between individuals with autism and typical controls in the inter-regional covariation of the insula, frontal area, caudate, amygdala, hippocampus, and PHG. Further, we highlight associations between covariation in density of the thalamus, putamen, precentral gyrus, frontal, parietal, and occipital lobes, and the cerebellum, and core autism symptoms, in particular repetitive behaviors and abnormal sensory behavior. Future studies may link our morphometry findings with data on brain function obtained from cognitive tests and/or functional and resting-state MRI, and with genomics data.

## Supplementary information

**Additional file 1:** **Subsection 1.** Acquisition parameters. **Subsection 2.** Demographic information of each schedule. **Subsection 3.** Sensitivity analyses. **Subsection 4.** Leave-one-out (LOO) validation of CCA results. **Subsection 5.** Group differences at voxel-wise gray matter volumes. **Subsection 6.** Independent components with case-control differences. **Subsection 7.** Robustness assessment of ICA model orders. **Subsection 8.** GLM results of the association between brain components and symptom profiles. **Subsection 9.** Components with highest loadings in CCA. **Subsection 10.** Uncorrected main CCA mode loadings of each component.

## Data Availability

Data collected in EU-AIMS LEAP are stored and curated at the central EU-AIMS database at the Pasteur Institute in Paris. The database is open to members of the wider scientific community upon request and submission of a paper and data analytic proposal.

## Abbreviations

ADHD: Attention Deficit Hyperactivity Disorder
ADI: Autism Diagnostic Interview-Revised
ADOS: Autism Diagnostic Observational Schedule 2
CCA: Canonical correlation analysis
DSM-IV: Diagnostic and Statistical Manual-IV
EU-AIMS: European Autism Interventions—A Multicentre Study for Developing New Medications
FDR: False discovery rate
FSIQ: Full-scale intelligence quotient
GLM: Generalized linear model
GM: Gray matter
IC: Independent component
ICA: Independent component analysis
ICD-10: International Statistical Classification of Diseases and Related Health Problems 10th Revision
ID: Intellectual disability
IFG: Inferior frontal gyrus
OFC: Orbitofrontal cortex
MRI: Magnetic resonance imaging
PHG: Parahippocampal gyrus
RBS: Repetitive Behavior Scale-Revised
RRB: Restricted and repetitive behaviors
SA: Social affect
SD: Standard deviation
SRS: Social Responsiveness Scale 2nd Edition
SSP: Short Sensory Profile
TD: Typically developing
TFCE: Threshold-Free Cluster Enhancement
VBM: Voxel-based morphometry
