# Supplementary material for: Gray matter covariations and core symptoms of autism: the EU-AIMS Longitudinal European Autism Project
Source: Mol Autism. 2020 Oct 30;11:86. doi: 10.1186/s13229-020-00389-4 (PMC7596954; doi:10.1186/s13229-020-00389-4)
Supplement: Supplementary file 1 — Additional file 1: Subsection 1. Acquisition parameters. Subsection 2. Demographic information of each schedule. Subsection 3. Sensitivity analyses. Subsection 4. Leave-one-out (LOO) validation of CCA results. Subsection 5. Group differences at voxel-wise gray matter volumes. Subsection 6. Independent components with case-control differences. Subsection 7. Robustness assessment of ICA model orders. Subsection 8. GLM results of the association between brain components and symptom profiles. Subsection 9. Components with highest loadings in CCA. Subsection 10. Uncorrected main CCA mode loadings of each component. [file 13229_2020_389_MOESM1_ESM.pdf]

Gray matter covariations and core symptoms in autism spectrum disorder. The EU-AIMS Longitudinal European Autism Project

Supplementary Information

Table of contents

1. Acquisition parameters 1
2. Demographic information of each schedule 2
3. Sensitivity analyses 3
4. Leave-one-out (LOO) validation of CCA results 7
5. Group differences at voxel-wise gray matter volumes 8
6. Independent components with case-control differences 9
7. Robustness assessment of ICA model orders 10
8. GLM results of the association between brain components and symptom profiles 12
9. Components with highest loadings in CCA 14
10. Uncorrected main CCA mode loadings of each component 15
References 16

1. Acquisition parameters

Table S1. Summary of acquisition parameters across sites

Site	Manufacturer	Model	Software Version	Acquisition sequence	Coverage	Slices	Thickness [mm]	Resolution [mm^3]	TR [s]	TE [ms]	FA [°]	FOV
Cambridge	Siemens	Verio	Syngo MR B17	Tfl3d1_ns	256*256	176	1.2	1.1*1.1*1.2	2.3	2.95	9	270
London	GE Medical systems	Discovery mr750	LX MR DV23.1_V02_1317.c	SAG ADNI GO ACC SPGR	256*256	196	1.2	1.1*1.1*1.2	7.31	3.02	11	270
Mannheim	Siemens	TimTrio	Syngo MR B17	MPRAGE ADNI	256*256	176	1.2	1.1*1.1*1.2	2.3	2.93	9	270
Nijmegen	Siemens	Skyra	Syngo MRD13	Tfl3d1_16ns	256*256	176	1.2	1.1*1.1*1.2	2.3	2.93	9	270
Rome	GE Medical systems	Signa HDxt	24/LX/MR HD16.0_V02_1131.a	SAG ADNI GO ACC SPGR	256*256	172	1.2	1.1*1.1*1.2	5.96	1.76	11	270
Utrecht	Philips Medical Systems	Achieva /Ingenia CX	3.2.3, 3.2.3.1	ADNI GO 2	256*256	170	1.2	1.1*1.1*1.2	6.76	3.1	9	270

TR: repetition time; TE: echo time; FA: flip angle; FOV: field of view.

2. Demographic information of each schedule

Participants were split into four schedules depending on their age and full-scale intelligence quotient (FSIQ). Schedule A included adults aged 18-30 years, Schedule B included adolescents aged 12-17 years, and Schedule C included children aged 6-11 years. In schedules A-C, all participants had a FSIQ in the typical range (FSIQ≥75). Lastly, Schedule D comprised adolescents and adults aged 12-30 years with mild intellectual disability (ID) (50≤FSIQ<75) (Table S2).

Table S2. Demographic information of participants in each schedule

	Schedule A		Schedule B		Schedule C		Schedule D	
Variable	Autism	TD	Autism	TD	Autism	TD	Autism	TD
N	114	85	115	85	73	59	45	23
Age,mean,[SD]	22.54 [3.25]	22.99 [3.34]	14.88 [1.77]	15.38 [1.74]	9.60 [1.44]	9.74 [1.49]	18.77 [4.41]	18.58 [4.39]
IQ,mean,[SD]								
Full-scale IQ	104 [15]	109 [13]	103 [15]	106 [13]	107 [14]	113 [13]	67 [5]	64 [9]
Performance IQ [a]	106 [16]	108 [15]	105 [18]	107 [15]	106 [14]	112 [15]	65 [10]	64 [11]
Verbal IQ [a]	103 [16]	109 [15]	100 [15]	104 [14]	107 [15]	113 [14]	69 [9]	64 [11]
Sex, N, [%]								
Male	82 [71.93]	56 [65.88]	90 [78.26]	59 [69.41]	52 [71.23]	36 [61.02]	29 [64.44]	12 [52.17]
Female	32 [28.07]	29 [34.12]	25 [21.74]	26 [30.59]	21 [28.77]	23 [38.98]	16 [35.56]	11 [47.83]
Site, N								
Cambridge	17	10	19	9	12	10	3	0
KCL	52	39	36	18	24	8	21	13
Mannheim	5	5	19	24	2	7	2	0
Nijmegen	25	12	29	26	27	22	19	10
Utrecht	16	19	12	9	9	13	1	0

TD, typically developed; SD, standard deviation; IQ, intelligence quotient.

[a] In Schedule C, there were 3 individuals with autism missing the performance and verbal IQ data.

3. Sensitivity analyses

3.1 FSIQ

To validate the results not being biased by low IQ participants we excluded the participants in Schedule D (FSIQ < 75) and performed an analogous 100-dimensional ICA decomposition, and automatic dimensional estimated factorizations (85 ICs) followed by post-hoc statistics again. In the 100-dimensional factorization case, we found the components with significant group effect were equivalent to IC10 and IC14 in the original analysis with the Schedule D participants included (Figure S1a), however, they did not survive FDR correction ($p<1.600\times10^{-5}$). Similarly, in the automatic dimensional factorization case, we found significant ICs corresponding to IC10 and IC14, however the IC corresponding to IC14 did not survive FDR correction ($p<7.531\times10^{-4}$) (Figure S1b).

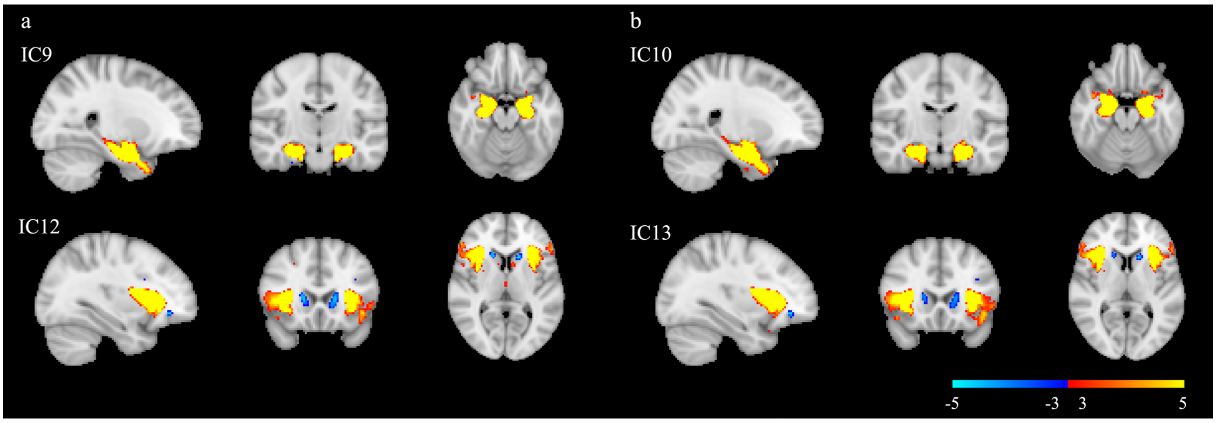

Figure S1. The components showed significant case-control differences. Panel **a** shows the components in **100** dimensional factorization **excluding** Schedule D participants. IC9 corresponds to IC14 in the 100 dimensional factorization including Schedule D participants (p=0.001), and IC12 corresponds to IC10 ($p=1.600\times10^{-5}$). Neither of these ICs survive FDR correction ($p<1.600\times10^{-5}$). Panel **b** shows the components in **automatic** dimensional factorization **excluding** Schedule D participants. IC10 corresponds to IC14 in 100 dimensional factorization including Schedule D participants ($p=7.531\times10^{-4}$), and IC13 ($p=3.830\times10^{-5}$) corresponds to IC10 that survived FDR correction ($p<7.531\times10^{-4}$). The component maps were thresholded at $3<|Z|<5$. IC, independent component.

3.2 ADHD symptoms

We ran additional group effect analyses (autism vs control) with ADHD symptoms as a covariate by using a dimensional score of ADHD symptoms (instead of the dummy code). The dimensional score was assessed by subscales of ADHD rating scale for symptoms of inattention and hyperactivity/impulsivity (Table S3). The result slightly differed from the analysis using the ADHD categorical score, where IC14 was found with significant group effect but IC10 was not. In the current analysis with ADHD dimensional score, component 14 was found with no significant group effect after FDR either (p=0.004). Since the continuous scores provide more variance of ADHD symptoms, this finding suggests IC14 (amygdala, hippocampus, and parahippocampal gyrus) is possibly confounded by the variance of comorbid ADHD symptoms.

Moreover, regarding the neural complexity of comorbidity with autism and ADHD, we excluded the participants that fulfilled the ADHD diagnosis on the ADHD rating scale and the participants with ADHD rating score unavailable, and then re-ran ICA and statistical analyses. The covariates in group effect analyses were the same as in the original main analyses. This resulted in 160 (46.1% of 347 participants) individuals with autism and 180 (71.4% of 252 participants) individuals with TD being included. Not surprisingly given

the significant reduction in numbers, we didn't find any significant result in these analyses. We attribute the non-significant output probably to the reduced statistical power.

Table S3. Demographic information of the 500 participants used to analyze the effect of comorbidity

**Demographic**	Autism, n = 299		TD, n = 201		$t/\mathcal{X}^2$	p value
	Mean	SD	Mean	SD		
Age, years [a]	16.79	5.59	16.77	5.59	-0.037	0.970
FSIQ [a]	99.74	19.05	105.31	17.26	3.397	p=0.001
	n	%	n	%		
Sex, male/female [b]	212/87	70.9/29.1	127/74	63.2/36.8	3.280	0.070
**Clinical**	n	%	n	%		
ADHD rating scale [b], with ADHD/without	139/160	46.5/53.5	21/180	10.4/89.6	71.750	p<0.001
	Mean	SD	Mean	SD		
ADHD rating scale [a] (dimensional score)	6.88	5.22	1.68	3.22		

TD, typically developing; SD, standard deviation; FSIQ, full-scale intelligence quotient; ADHD, attention deficit hyperactivity disorder.

[a] Statistical differences were assessed by two sample *t*-test.

[b] Statistical difference was examined by the chi-square test.

3.3 Age

As the rate of gray matter development may not be linear across a wide age range (1), potential effects of age squared, age-by-group, and age squared-by-group interactions should be accounted for in the statistical model. Accordingly, to avoid overfitting the model and acquire an optimal mode of the statistical model, we additionally ran a full model with these variables on the components found significant with group effect, and stepwise removed the age relating terms depending on their contribution to the model fit. After comparing the goodness of fits between the models, we hence acquired an appropriate model for case-control difference analyses. In the main results, we found that autism group showed significant difference of IC10 and IC14 from TD group. Therefore, we did the above mentioned analyses on these two components separately. We observed that in the analysis of IC10 the age squared variable did improve the fit of statistical model, while none of the age relating variables enhanced the fit of model for IC14 (Table S4). Consequently, we additionally added the age squared variable into the original model, and then found similar outputs as the main results. That is, we found IC10 (b=-0.147, p=8.996X10-5) and IC14 (b=-0.132, p=5.465x10-4) remaining significance contributors to the group effects (FDR corrected, p<7.751x10-4).

Table S4. The statistical values for model comparison using analysis-of-variance

Models	F	p	result
IC10			
Model 1 vs model 2	0.040	0.841	Keep model 2
Model 2 vs model 3	2.138	0.144	Keep model 3
Model 3 vs model 4	6.605	0.010	**Keep model 3**
IC14			
Model 1 vs model 2	0.547	0.046	Keep model 2
Model 2 vs model 3	0.051	0.821	Keep model 3

Model 3 vs model 4	0.063	0.802	**Keep model 4**

Model 1: component ~ group + age + age$^{\wedge 2}$ + age*group + age$^{\wedge 2}$*group + sex + FSIQ + sites; Model 2: component ~ group + age + age$^{\wedge 2}$ + age*group + sex + FSIQ + sites; Model 3: component ~ group + age + age$^{\wedge 2}$ + sex + FSIQ + sites; Model 4 (original model): component ~ group + age + sex + FSIQ + sites.
IC, independent component.

3.4 Sex-by-group interaction

As sex ratio is uneven between autism and control group, we accounted for the possible effect of sex-by-group interaction. Therefore, we repeated the analyses similarly to the Age section to find an optimal mode of the statistical model. As a result, adding sex-by-group did not significantly improve the fit of the model (Table S5). Therefore, we kept the original model.

Table S5. The statistical values for model comparison using analysis-of-variance

Models	F	p	result
IC10			
Model 1 vs model 2	0.484	0.487	**Keep model 2**
IC14			
Model 1 vs model 2	0.002	0.961	**Keep model 2**

Model 1: component ~ group + age + sex + sex*group + FSIQ + sites; Model 2 (original model): component ~ group + age + sex + FSIQ + sites.
IC, independent component.

3.5 Anxiety and depression symptoms

In addition to ADHD rating scale, we used the Development and Well-Being Assessment (DAWBA) anxiety and depression prediction scores to investigate their separate effects on group differences of structural covariance (2). In DAWBA, each scale reflects six levels of predication (i.e., from ~0.1% to >70%) of the probability of meeting clinically relevant diagnostic criteria for a disorder. The anxiety prediction score reflects the highest risk of an individual across a group of anxiety disorders (obsessive-compulsive disorder (OCD), generalized anxiety, panic disorder, agoraphobia, PTSD, separation anxiety, social phobia, and specific phobia). The depression prediction score was generated for major depression. The information for the participants with available score was shown in Table S6.

We used anxiety and depression scores as additional covariates in the statistical model separately. In the analyses including the anxiety score (N=494), no significant group effects found, while the component with smallest p value is component 14 ($p=7.289 \times 10^{-4}$), which was found with significant group effect in original analyses, comprising amygdala, hippocampus, and parahippocampal gyrus. In the other analyses including depression score (N=446), component 10 (insula, frontal areas, and caudate) was found significantly different in autism group ($p=1.302 \times 10^{-4}$, FDR corrected, $p<8.99 \times 10^{-4}$).

Although there were no significant results remaining in the analysis where anxiety score was accounted for, the p values are relatively small, and it's meaningful that the two components demonstrate differences when taking the anxiety and depression comorbidities into account. Previous studies showed individuals with autism and individuals with anxiety both involved in the structural differences in inferior frontal gyrus, insula, striatum and amygdala (3, 4), which indicates that IC10 probably reflects shared variances related to autism and anxiety on structural covariance. Major depression was formerly reported related to gray matter alterations in amygdala, hippocampus (e.g. (5)), which may suggest structural covariance in IC14 reflect shared variances between autism and depression symptoms.

Table S6. Sample characteristics of DAWBA and Medication

	Autism, n = 286		TD, n = 208		range
	Mean	SD	Mean	SD	
DAWBA anxiety	2.48	1.33	1.19	0.85	0 ~ 5
	Autism, n = 299		TD, n = 185		
	Mean	SD	Mean	SD	
DAWBA depression	0.96	1.29	0.39	0.85	0 ~ 5
	Autism, n = 347		TD, n = 250		
	n	%	n	%	
Medication use yes/no	218/129	62.8/37.2	231/19	92.4/7.6	

SD, standard deviation; DAWBA, Development and Well-Being Assessment; TD, typically developing."

3.6 Medication use

Since there is no strong and specific medication for autism, along with the high rate of comorbidity, medication use is heterogeneous in its nature (e.g. antidepressant, antipsychotic, sedatives, and medication treating ADHD). Therefore, we used a categorical score as a covariate to indicate whether the individuals take psychotropic medication (acting on the nervous system [data available on N=597, N=148 on psychotropic medication, Table S6). After regressing out medication use, we found that IC14 was not significant after FDR correction (p=0.003), while IC10 still showed significant group differences ($p=1.162x10^{-5}$, FDR corrected, $p<4.406x10^{-4}$). This is partly in line with findings that suggest the volume of subcortical area is associated with medication use (6, 7).

Unfortunately, as unknown medication use could be confounding, and the various medications that individuals use are complex, the results of adding detailed medication use as a covariate would be difficult to interpret at best and likely underpowered to enable specific conclusions.

3.7 Sample homogeneity

Considering potential diagnostic difference of data quality that might influence the results, we checked the group differences of mean correlation from sample homogeneity measure while regressing out additional confounders; sex, site, FSIQ and age. Moreover, we also added it as an additional covariate into the statistical model for detecting group effect on structural covariance. We found that the homogeneity of gray matter images in autism group (mean: 0.878, standard deviation: 0.007) had no significant difference from TD group (mean: 0.879, standard deviation: 0.006; b=-0.051, p=0.176). Furthermore, as a covariate, the image quality had no significant effect on the main results we found. That is, IC10 was still found significantly associated with autism group ($p=1.788x10^{-4}$), while the group effect of IC14 was found at FDR threshold (p=0.001).

4. Reproducibility of CCA results

To assess the reproducibility of CCA results, we employed a leave-one-out (LOO) approach by randomly resampling subsets of the sample with participant number from 50 to 325 (ADI&ADOS)/194 (SRS&RBS&SSP), and repeating each LOO analysis 50 times. In each subset, we separately correlated the main mode weights of brain loadings and behavior profiles, which were generated from LOO analysis of CCA, with the weights of the original main mode. We then used the mean and standard deviation of r values to evaluate the reproducibility of CCA in different sample sizes. In CCA_1 (ADI&ADOS), the weights of the main CCA mode of each leave-one-out analysis correlated on average above 0.94 with the weights of original main CCA mode in brain loadings and above 0.95 in behavior profiles when the sample was bigger than 122. In CCA_2 (SRS&RBS&SSP), the weights of the main CCA mode related on average above 0.92 in brain loadings and above 0.96 in behavior profiles when the sample was bigger than 111. Both CCA analyses are no reproducible for sample sizes smaller than (approximately) 100 subjects. (Figure S2).

a

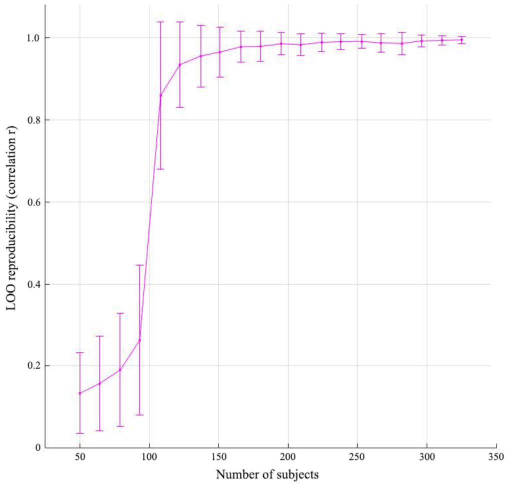

LOO validation of brain loadings in the main CCA mode (ADI&ADOS)

b

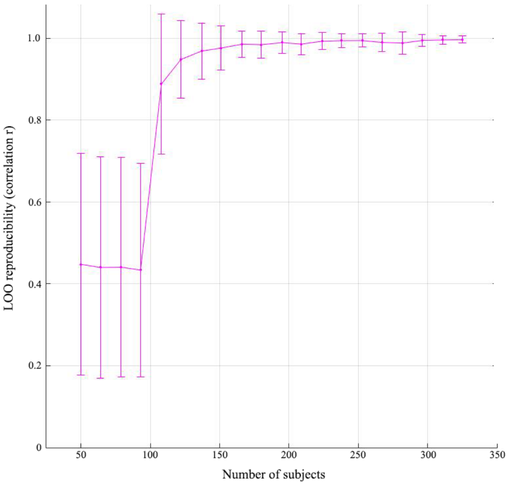

LOO validation of behavior profiles in the main CCA mode (ADI&ADOS)

c

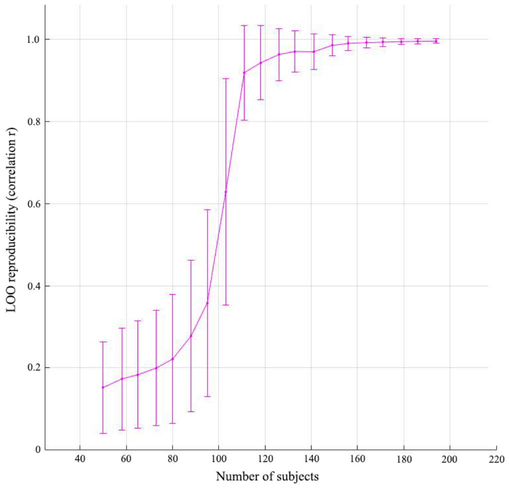

LOO validation of brain loadings in the main CCA mode (SRS&RBS&SSP)

d

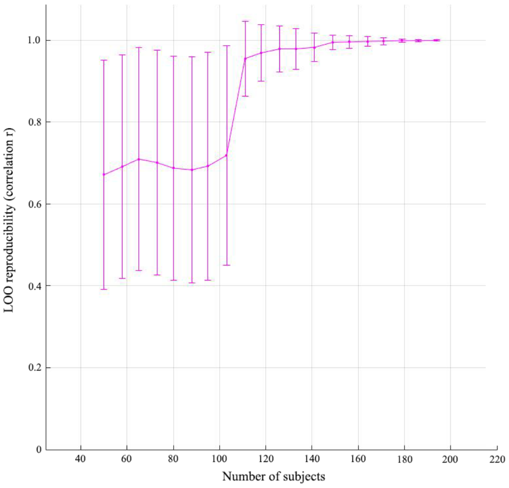

LOO validation of behavior profiles in the main CCA mode (SRS&RBS&SSP)

Figure S2. LOO validation of the main CCA modes in both CCA. (a, c) display the reproducibility of brain

components of the main CCA mode related with ADI and ADOS (a), and with SRS, RBS, and SSP (c). (b, d) show the reproducibility of symptom profiles in each main CCA mode. LOO, leave-one-out; CCA, canonical correlation analysis; ADI, Autism Diagnostic Interview-Revised; ADOS, Autism Diagnostic Observational Schedule 2; SRS, Social Responsiveness Scale 2nd Edition; RBS, Repetitive Behavior Scale-Revised; SSP, Short Sensory Profile.

5. Group differences at voxel-wise gray matter volumes

The standard mass-univariate GLM analysis of the VBM data comparing cases and controls did not show significant group differences for voxel-wise GM densities. Figure S3 presents each voxel t-statistics and it is thresholded at uncorrected $p<0.05$.

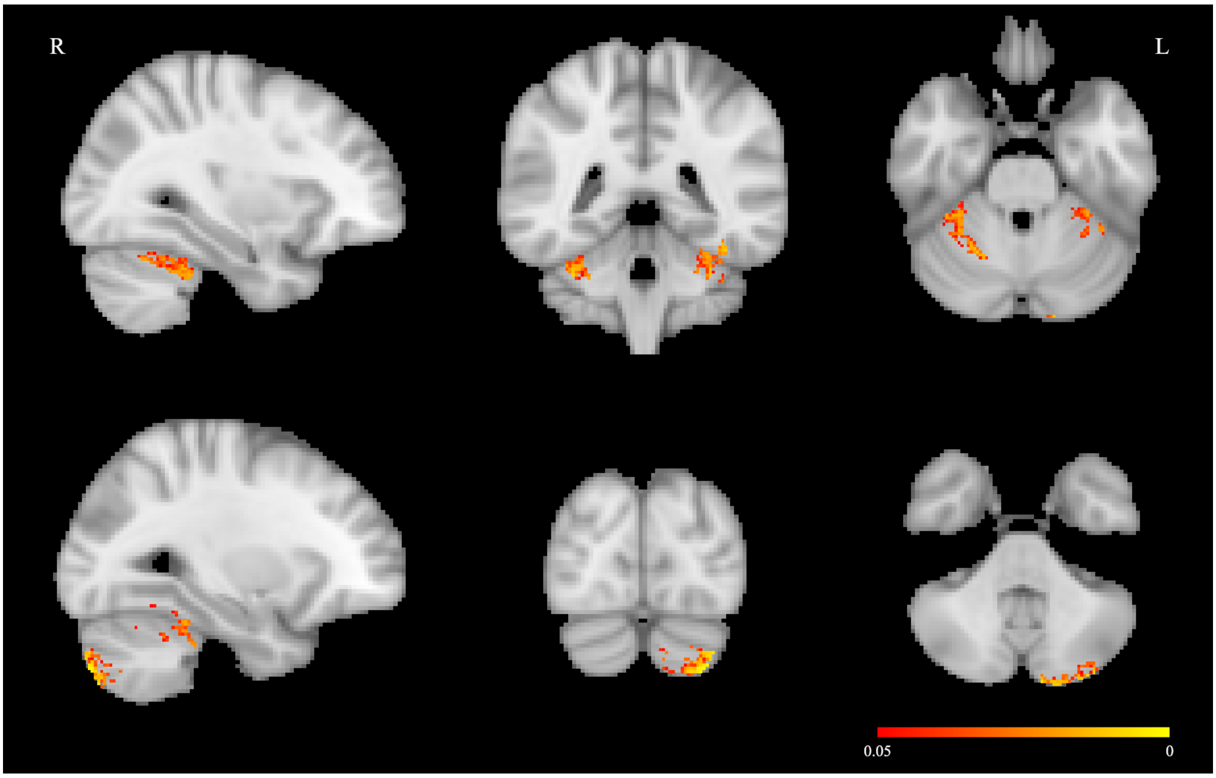

Figure S3. Results of case-control differences of voxel-wise gray matter densities ($p<0.05$, uncorrected).

6. Independent components with case-control differences

Nine independent components (ICs) showed case-control differences ($p<0.05$, i.e. IC10, IC13, IC14, IC15, IC23, IC28, IC31, IC48, and IC 99, Figure S4), among which IC10 (β=-0.175, $p=8.850\times10^{-5}$) and IC14 (β=-0.152, $p=5.450\times10^{-4}$) survived FDR correction ($p<8.072\text{x}10^{-4}$).

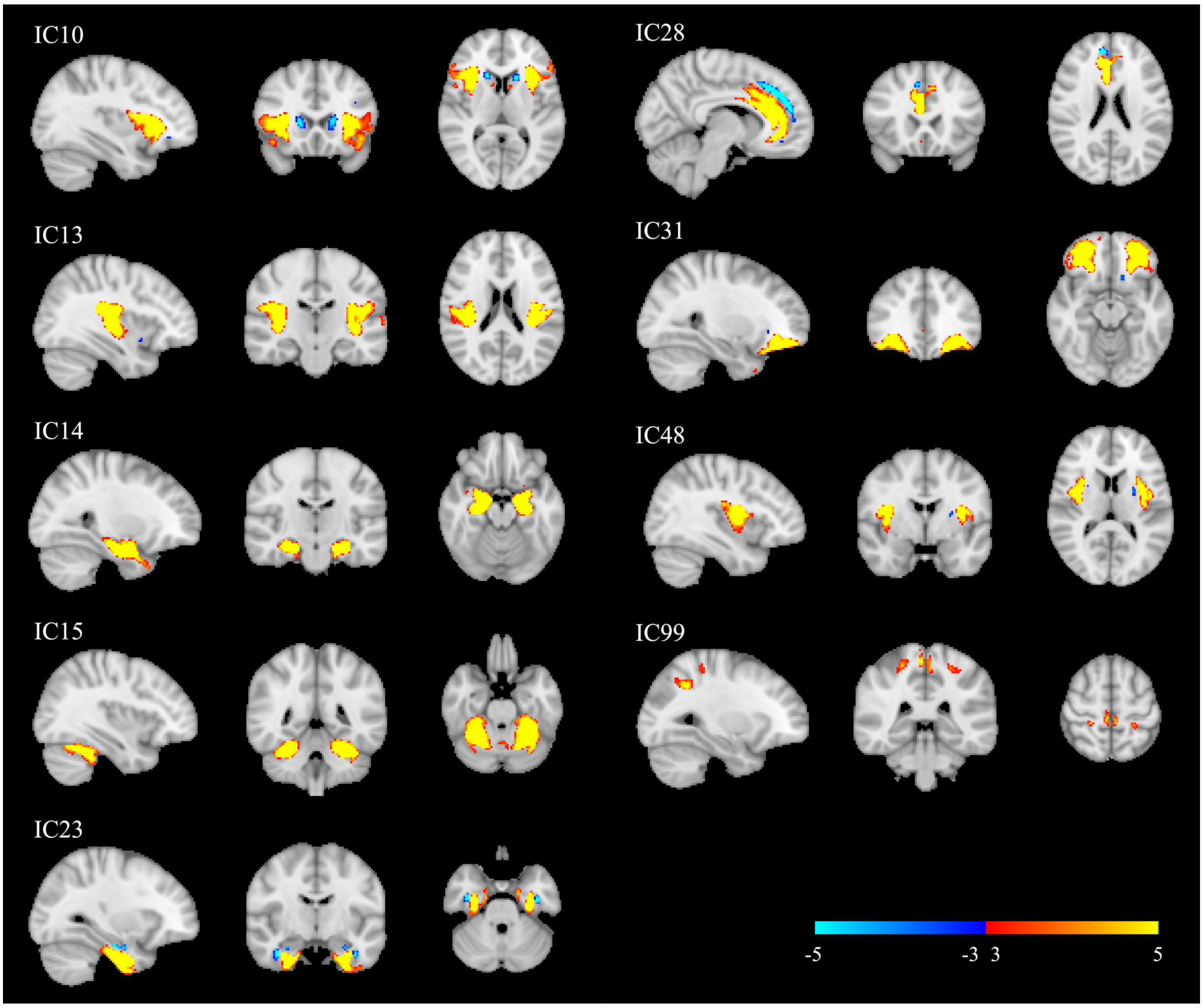

Figure S4. The components showed significant case-control differences ($p<0.05$, uncorrected). The component maps were thresholded at $3<|Z|<5$. IC, independent component.

7. Robustness assessment of the ICA model orders

To assess the reproducibility of components IC10 and IC14 obtained from the mainly reported 100-dimensional decomposition, we first correlated the participant loadings from the 100-dimensional factorization with the participant loadings obtained from an alternative factorization. This allowed us to identify the two components from the alternative factorization that are more strongly correlated to IC10 and IC14 respectively, and evaluate then the spatial reproducibility achieved at the alternative factorization. Post-hoc statistics, for automatic estimation (91 ICs) and the 50-dimensional IC analysis factorization showed that the composition of components with significant group effects were similar to the original analysis with 100 components. Significant results of both dimensional factorizations are almost equivalent to the IC10 (automatic dimension: $p=2.109\times10^{-4}$, 50 dimension: $p=0.002$) and IC15 (automatic dimension: $p=3.557\times10^{-4}$, 50 dimension: $p=2.778\times10^{-4}$) in the analysis of 100-dimensional factorization, however, the ICs corresponding to IC14 in automatic dimension ($p=4.733\times10^{-4}$) and 50 dimensions ($p=0.003$) did not survive FDR correction (automatic dimension: $p<4.733\times10^{-4}$; 50 dimension factorization: $p<0.003$) (Figure S5).

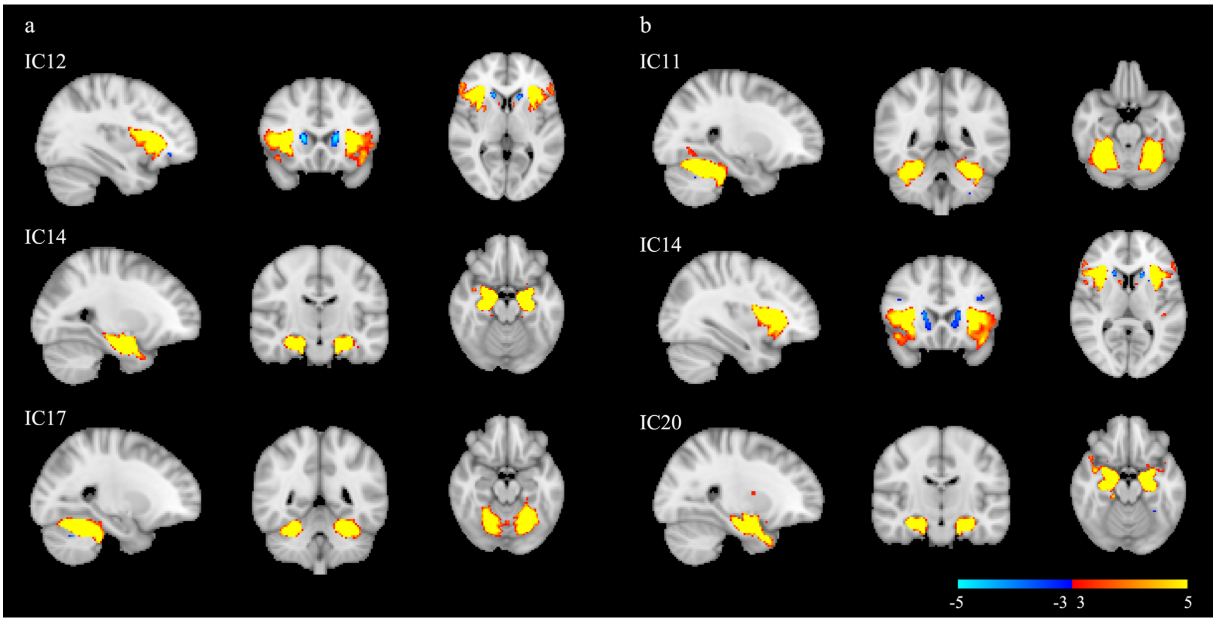

Figure S5. The components showed significant case-control differences. Panel **a** shows the components in **automatic** dimensional factorization. IC12 ($p=2.109\times10^{-4}$), corresponding to IC10 in 100-dimensional factorization, and IC17 ($p=3.557\times10^{-4}$) survived multiple comparison correction ($p<4.733\times10^{-4}$). IC14 ($p=4.733\times10^{-4}$), corresponding to the IC14 in 100-dimensional factorization did not survive correction. Panel **b** shows the components in 50-dimensional factorization. IC11 ($p=2.778\times10^{-4}$), and IC14 ($p=0.002$), corresponding to IC10 in 100-dimensional factorization, survived multiple comparison correction ($p<0.003$). IC20 ($p=0.003$), corresponding to IC14 in 100-dimensional factorization did not survive correction. The component maps were thresholded at $3<|Z|<5$. IC, independent component.

Table S7. Summary of robustness assessment of ICA results (correlation results)

conditions		corresponding IC	participant loadings		spatial maps	
			r	p	r	p
IC10	automatic dimension	IC12	0.990	p<0.001	0.979	p<0.001
	50 dimensions	IC14	0.941	p<0.001	0.879	p<0.001
IC14	automatic dimension	IC14	0.994	p<0.001	0.990	p<0.001
	50 dimensions	IC20	0.927	p<0.001	0.870	p<0.001

IC, independent component.

8. GLM results of the association between brain components and symptom profiles

Table S8. GLM results of the association between brain components and symptom profiles in autism group (uncorrected, $p<0.05$)

component	ADI						ADOS				SRS [a]		RBS		SSP	
	social		communication		RRB		social affect		RRB							
	b	p	b	p	b	p	b	p	b	p	b	p	b	p	b	p
2	-0.150	0.009														
3							-0.194	6.169×10^{-4}								
5							-0.147	0.007								
6	-0.120	0.046														
9					0.120	0.032										
12	-0.124	0.016					-0.112	0.033								
14									-0.133	0.022						
15									0.107	0.049						
21			0.108	0.048												
24									0.114	0.035						
27											0.116	0.030				
31									-0.130	0.010						
33	-0.108	0.039														
40													0.110	0.035		
41	-0.131	0.016					-0.171	0.002			-0.121	0.031				
42											0.108	0.046				
44	0.141	0.006	0.110	0.029												
51															0.130	0.044
57					-0.123	0.048										
59									0.167	0.001						
61					-0.120	0.023										
63							-0.138	0.028								

Table S8. GLM results of the association between brain components and symptom profiles in autism group (*p*<0.05, continued)

component	ADI						ADOS				SRS [a]		RBS		SSP	
	social		communication		RRB		social affect		RRB							
	b	p	b	p	b	p	b	p	b	p	b	p	b	p	b	p
65					-0.112	0.039										
69															-0.142	0.022
82															-0.212	4.169x10-4
89			-0.108	0.038												
90													-0.125	0.032		
95	-0.131	0.015														
97															-0.125	0.038
98															-0.127	0.048
100													0.112	0.034	-0.170	0.005

The association analyses were only performed in autism group. ADI, Autism Diagnostic Interview-Revised; RRB, restricted, repetitive behaviors; ADOS, Autism Diagnostic Observational Schedule 2; SRS, Social Responsiveness Scale 2nd Edition; RBS, Repetitive Behavior Scale-Revised; SSP, Short Sensory Profile.

[a] We used SRS parent T-scores.

9. Components with highest loadings in CCA

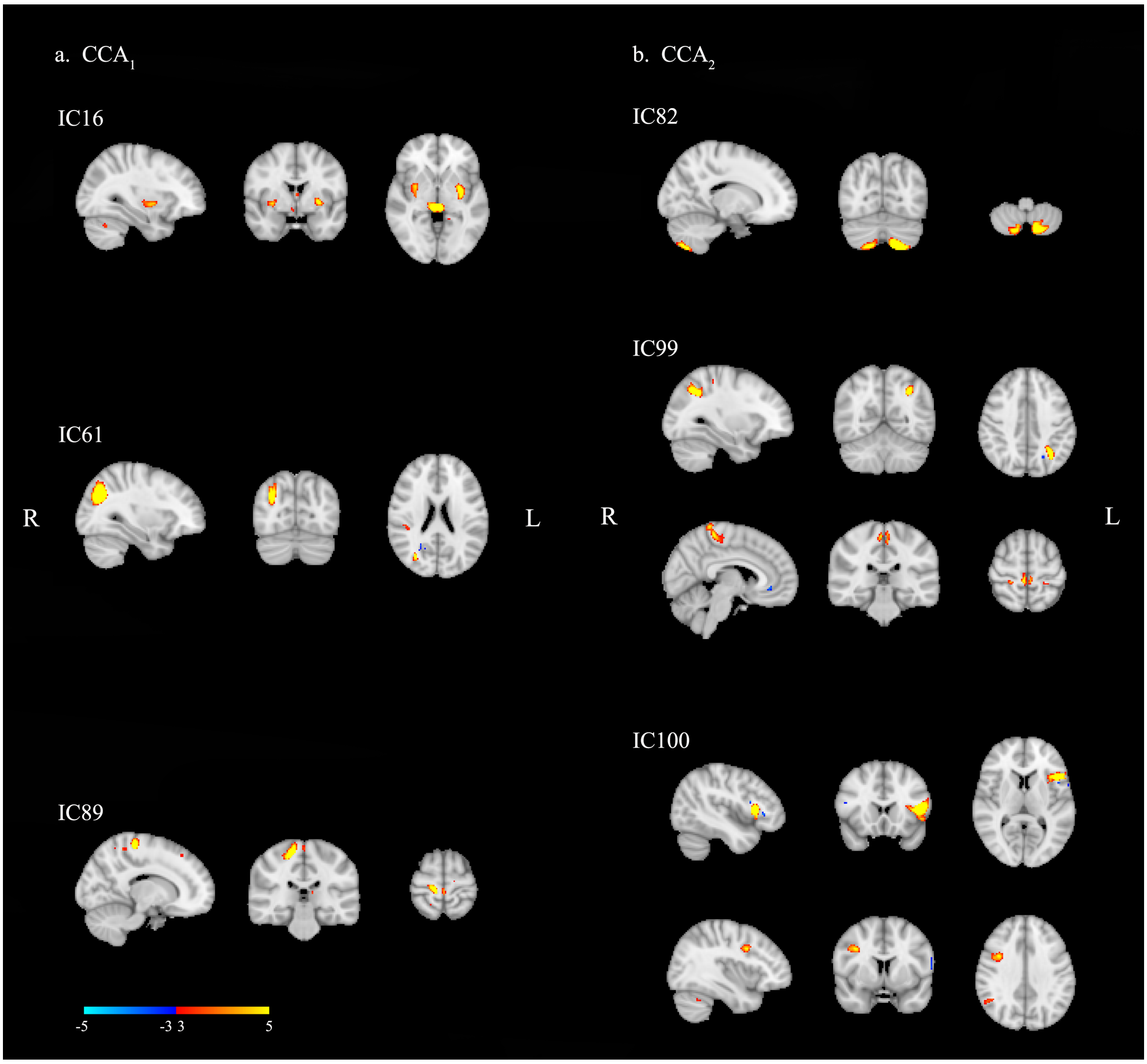

Figure S6. Components with highest loadings in CCA. Panel **a** shows the three components with highest loadings in CCA_1 (correlation with ADI and ADOS subscales). Panel **b** shows the three components with highest loadings in CCA_2 (correlation with SRS, RBS, and SSP). The component maps were thresholded at $3<|Z|<5$.

10. Uncorrected main CCA mode loadings of each component

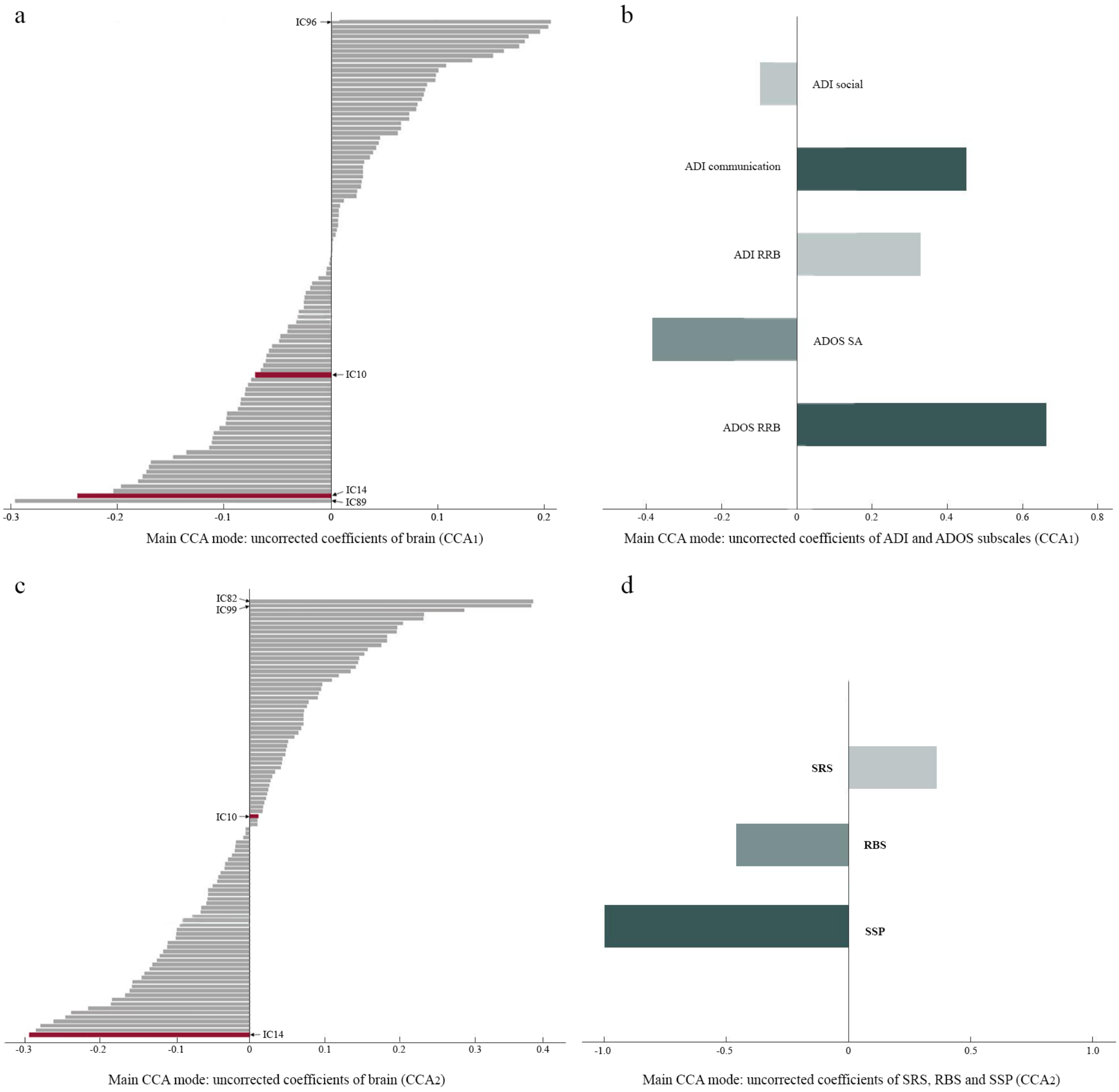

Figure S7. The top row shows **uncorrected** canonical coefficients (uncorrected weights) of the main CCA mode for the CCA_1 analyses (ADI&ADOS), and the bottom row for the CCA_2 analyses (SRS&RBS&SSP). Panels (a, c) display the degree that each brain component contributed to the main CCA mode in each analysis with respect to the uncorrected canonical coefficients. The two components with significant group effects are displayed in red. Panels (b, d) display the degree that each symptom profile contributes to each analysis. Among the uncorrected coefficients, IC14 ranks third among the 100 components when correlating to ADI and ADOS (a), and it ranks fourth in the CCA with SRS, RBS, and SSP (c). CCA, canonical correlation analysis; ADI, Autism Diagnostic Interview-Revised; ADOS, Autism Diagnostic Observational Schedule 2; SRS, Social Responsiveness Scale 2nd Edition; RBS, Repetitive Behavior Scale-Revised; SSP, Short Sensory Profile; IC, independent component.

References

1. Ecker C, Bookheimer SY, Murphy DG. Neuroimaging in autism spectrum disorder: brain structure and function across the lifespan. Lancet Neurol. 2015;14(11):1121-34.

2. Goodman R, Ford T, Richards H, Gatward R, Meltzer H. The Development and Well-Being Assessment: description and initial validation of an integrated assessment of child and adolescent psychopathology. J Child Psychol Psychiatry. 2000;41(5):645-55.

3. Yin S, Hong SK, Di Martino A, Milham MP, Park BY, Benkarim O, et al. Shared and distinct patterns of atypical cortical morphometry in children with autism and anxiety.

4. Carlisi CO, Norman LJ, Lukito SS, Radua J, Mataix-Cols D, Rubia K. Comparative Multimodal Meta-analysis of Structural and Functional Brain Abnormalities in Autism Spectrum Disorder and Obsessive-Compulsive Disorder. Biol Psychiatry. 2017;82(2):83-102.

5. Lee HY, Tae WS, Yoon HK, Lee BT, Paik JW, Son KR, et al. Demonstration of decreased gray matter concentration in the midbrain encompassing the dorsal raphe nucleus and the limbic subcortical regions in major depressive disorder: an optimized voxel-based morphometry study. J Affect Disord. 2011;133(1-2):128-36.

6. Nakao T, Radua J, Rubia K, Mataix-Cols D. Gray matter volume abnormalities in ADHD: voxel-based meta-analysis exploring the effects of age and stimulant medication. Am J Psychiatry. 2011;168(11):1154-63.

7. Hashimoto N, Ito YM, Okada N, Yamamori H, Yasuda Y, Fujimoto M, et al. The effect of duration of illness and antipsychotics on subcortical volumes in schizophrenia: Analysis of 778 subjects. Neuroimage Clin. 2018;17:563-9.
